# Compost Barns: A Bibliometric Analysis

**DOI:** 10.3390/ani12192492

**Published:** 2022-09-20

**Authors:** Gustavo Guimaraes Bessa Santos Silva, Patrícia Ferreira Ponciano Ferraz, Flávio Alves Damasceno, Maria Luísa Appendino Nunes Zotti, Matteo Barbari

**Affiliations:** 1 Department of Animal Science, Federal University of Lavras (UFLA), Lavras 37200-900, Brazil; 2 Department of Agricultural Engineering, Federal University of Lavras (UFLA), Lavras 37200-900, Brazil; 3 Department of Engineering, Federal University of Lavras (UFLA), Lavras 37200-900, Brazil; 4 Department of Animal Science, Santa Catarina State University (UDESC), Chapecó 89815-630, Brazil; 5 Department of Agriculture, Food, Environment and Forestry, University of Firenze, 13-50145 Firenze, Italy

**Keywords:** dairy cattle, dairy cow, compost-bedded pack barn

## Abstract

**Simple Summary:**

In the dairy farming system that uses compost barns, animals remain in a large area covered with organic material and are free to move and express their behaviors in a more natural way. The compost barn system has become popular in recent years because it provides greater well-being and quality of life, favoring animal productivity and longevity. The aim of this paper is to develop a bibliometric analysis to evaluate scientific studies about compost barn systems. A total of 108 databases, considering articles and literature reviews obtained from the Scopus and Web of Science databases were considered for this analysis. After standardizing the data, the resulting spreadsheet was analyzed with VOSviewer software. The keywords most used by the authors were “compost-bedded pack barn”, “dairy cow”, and “dairy cattle”. The most relevant countries, journals, institutions, researchers, and co-citation networks to compost barn research were highlighted. The analysis confirmed a significant interest in the spatial variation in the sheds and their relationship with milk quality, heat stress, and animal welfare. This paper provides a great contribution related to the scientific evolution and the research and publishing tendencies of studies on the compost barn animal housing system.

**Abstract:**

The objective of this study was to evaluate the main scientific studies addressing the topic of compost barns in recent years, highlighting the main journals, authors, countries, organizations, and keywords associated with the publications and trends in this type of research through a bibliometric analysis. For this analysis, publications (articles and literature reviews) addressing compost barns were obtained from the Scopus and Web of Science databases. A total of 108 studies published between 2007 and April 2022 were included. A greater number of publications was observed starting in 2017, with 2021 having the largest number of publications. The Journal of Dairy Science was the most highly cited journal. Marcia I. Endres was the author with the greatest academic impact. The most influential country was the United States, followed by Brazil. Among the organizations that have published studies on compost barns, the Federal University of Lavras, and the University of Florence had the largest numbers of publications. In conclusion, this bibliometric analysis allowed us to evaluate the scientific evolution, research, and publishing tendencies of studies on the compost barn animal housing system, and the results make it possible to deduce current trends in scientific research and publications.

## 1. Introduction

Worldwide, the use of confinement systems for dairy cattle has grown in recent decades [1]. The choice of rearing system is an important decision for milk producers because animals will spend most of their time in these systems. In addition, production systems have a strong influence on productivity, health, milk quality, reproduction, animal welfare, and farm profitability [2].

In recent decades, different housing systems have been developed, driven mainly by technical innovations imposed by changes in cow requirements, farmers’ demands, and social and environmental impacts [1]. Thus, due to the growing international demand for animal welfare, housing systems that provide space for cows to express their natural behaviors are becoming increasingly popular among producers [3]. The search for confinement systems that ensure the maintenance of satisfactory results in dairy cattle production has grown worldwide in recent decades. Among the intensive production systems used for the confinement of dairy cows in Brazil, the compost barn system has aroused the interest of dairy farmers [4].

Compost barns, also known as compost-bedded pack barns, are an alternative system of animal housing developed by a producer in Minnesota (USA) in 2001, in which animals are collectively housed in a large area covered with litter [5]. In general, the bedding area is surrounded by a masonry wall that retains the bedding material and animal waste for approximately 6 to 12 months [6]. In other cases, the soil in the litter area is removed, thus avoiding the need to build walls. In this system, the animals remain in a large area covered with organic material, where they are free to move and express their behavior in a more natural way. In addition, the barns have a feeding corridor, where the animals feed, a feed track, through which feed is distributed, and an access corridor, through which the cows have access to the milking parlor [7].

Black et al. [4] reported that housing animals in well-managed compost barns, in addition to resulting in healthier cows with better hygiene scores, increased milk production and reduced somatic cell count (SCC), the calving interval, and the service period when transitioning animals to the compost barn.

Because it is a relatively new housing system, analyzing the literature on compost barns is of great importance, as it will allow a synthesis of all the knowledge already available. Conducting systematic reviews, for example, allows us to select studies on a particular topic or area of interest, highlighting what is already known and exposing future opportunities [8,9].

Scientific mapping aims to create a representation of the structure of a research area by dividing elements (i.e., documents, authors, journals, or keywords) into different groups using a quantitative approach, resulting in a description and evaluation of published studies and the ability to monitor trends. This methodology is helpful in literature reviews even before reading begins, guiding researchers to the most influential work and mapping the research field without subjective bias [10].

Thus, the objective of this study was to evaluate the evolution of publications on compost barns over time; identify the main journals, authors, countries, and relevant organizations associated with the publications; and determine the keywords most used in publications and research trends on this type of accommodation through a bibliometric analysis.

## 2. Materials and Methods

**Figure 1:**
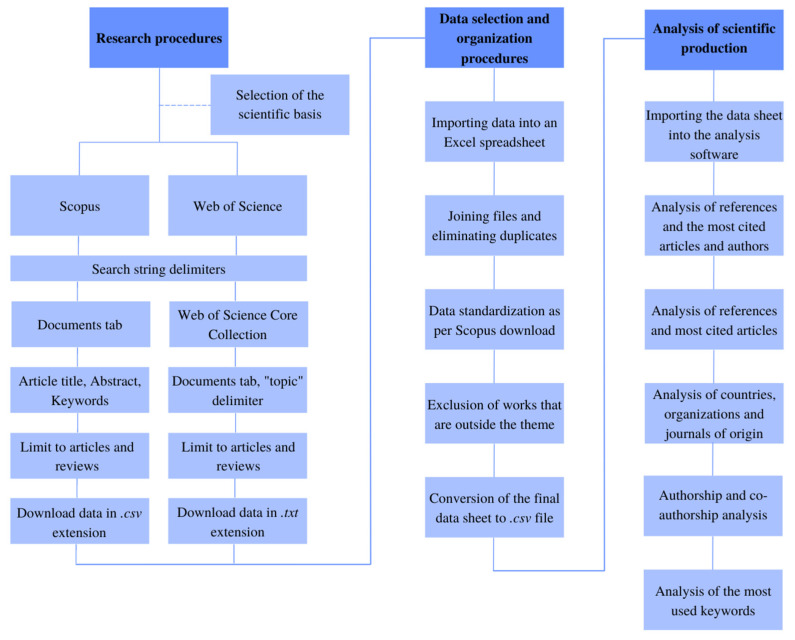
Systematization of the process for carrying out the bibliometric analysis.

The evolution of studies on dairy cattle housing in compost barn facilities was evaluated by means of a bibliometric analysis using scientific mapping. The general workflow in a scientific mapping analysis is divided into data retrieval, pre-processing, network extraction, normalization, mapping, analysis, and creation of a visual representation, which an analyst can later interpret and draw conclusions from [11,12,13]. The procedures used in this study are described in Figure 1, and the step-by-step procedure will be discussed in the subsequent subsections.

### 2.1. Research Procedure

Aiming to obtain a representative number of studies, the scientific databases selected for this research were Scopus and Web of Science. The choice and identification of these databases is justified because they are the largest databases of scientific literature worldwide [14].

Before performing a bibliometric analysis, it is important to understand the topic that will be researched to define the keywords, which should address the main research topic. In the second step, we defined the search filters, which were research time (year), area and subarea, and decided whether to include only articles published in journals or annals of events and publications in various languages [15].

The object of interest in this study is a specific type of housing for dairy cattle, the compost barn; thus, observing some terms used in studies on this subject contributed to the delimitation of the search terms used to cover the largest number of publications. The key terms used for database searches were “compost barn”, “compost bedded”, “compost-bedded” and “compost bedding”. Only publications containing the key terms in the title, abstract, or keywords were considered.

For the Scopus database, in the document search tab, the string used was article title, abstract, keywords ({compost barn} OR {compost bedded} OR {compost-bedded} OR {compost bedding}). In the Web of Science, the strings were searched for in the main collection of the Web of Science though the documents tab (topic (compost-barn OR compost-bedded OR compost-bedding)). In both databases, the documents were limited to only articles and reviews. Brackets were used to return exact match words, while the Boolean operator OR was used to find records that contained one of the terms separated by this operator. However, there were no differences in the results when the terms were searched in the plural or singular, as this combination of words returned the highest number of results.

The search conducted in the Scopus database resulted in 278 studies, with 225 articles and 31 literature reviews. Thus, 256 publications (articles and reviews) were selected, and the data were downloaded with a *.csv* extension. The Web of Science resulted in 140 publications, including 113 articles and 7 literature reviews (120 suitable publications), which were downloaded with a *.txt* extension.

### 2.2. Selection Procedures and Data Organization

The data selection and organization process consisted of reviewing the data obtained. For this step, the spreadsheets obtained from the databases were imported into Excel, where the titles and DOI codes were aligned to eliminate duplicate studies. Subsequently, by carefully reading the abstracts, the publications that diverged from the topic were eliminated, leaving 108 studies, of which 65 were in both databases, 38 were exclusive to Scopus and five were exclusive to the Web of Science database.

These data were organized and standardized in another spreadsheet according to the model of the spreadsheet downloaded from Scopus. This standardization is necessary because the Web of Science data include different codes that reference data. Thus, the next step was the conversion of this spreadsheet into a *.csv* file for later use in the bibliometric analysis software.

### 2.3. Analysis of Scientific Production

VOSviewer, which is a program developed to build, visualize, and explore bibliometric maps, was used for the identification and analysis of bibliometric networks [16].

The VOSviewer mapping and clustering results can be saved as map and network files. These file types can be viewed and edited using a text editor or spreadsheet program (for example, Excel) [17]. Therefore, this software was used to construct bibliometric maps of author co-citations, map associated organizations, identify the keywords most used by the authors and determine the tendency to use these words over time. After extracting the files from VOSviewer as data spreadsheets, it was possible to construct a citation graph of the countries and tables of publications, authors, and sources of the most relevant publications.

## 3. Results and Discussion

### 3.1. Evolution of Publications

**Figure 2:**
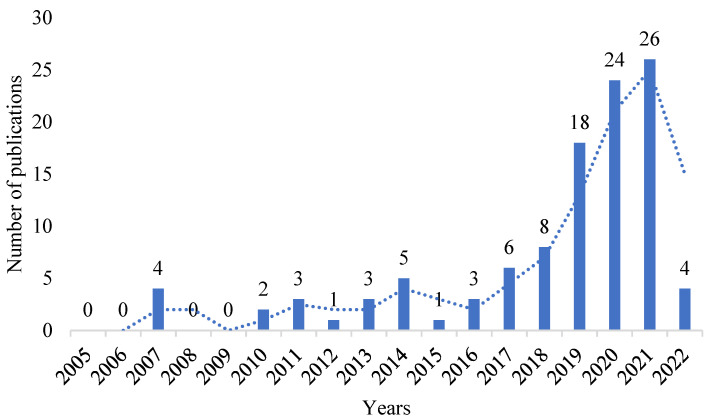
Evolution of research publications on compost barn from 2007 to 2022/April.

The bibliometric analysis included 108 publications on the compost barn cattle farming system published from 2007 to April 2022. The distribution of these publications over the study period is shown in Figure 2, which illustrates the number of publications per year.

Research on this type of system is very recent, since the first studies found in the databases of journal articles are from 2007. The pioneers in research on the compost barn system were Barberg et al. [5,18], Endres and Barberg [19], and Janni et al. [6].

Barberg et al. [18] conducted a descriptive study of 12 sheds that used the compost barn system, describing the layout and dimensions of the sheds, characterizing the bedding material, and observing the management practices of the facilities. In the study by Barberg et al. [5], in addition to describing the facilities and management practices used for the herds, the authors also evaluated the welfare, performance, and udder health of the animals before and after a change from the conventional system to the compost barn housing system and assessed the level of producer satisfaction in relation to the newly adopted system.

Endres and Barberg [19] measured the laying behavior and social interactions of lactating cows housed in a compost barn system and investigated the association between the temperature and humidity index (THI) and the laying behavior of these cows.

Janni et al. [6] also scientifically described the compost barn system, discussing recommendations for the layout and management of the composting bed, and concluded that this type of housing required further research.

Subsequent studies were published only in 2010 by Shane et al. [20,21]; these studies addressed one of the major bottlenecks of the compost barn system, namely, the cost and availability of bedding material. These authors described alternative bedding materials for partial or total replacement of sawdust in their sheds.

Thus, over time, the number of adherents to the system increased, and there are reports of its adoption in several countries, including the United States, especially in the Midwest and Northeast, Japan, China, Germany, Italy, the Netherlands, Israel, Denmark and, recently, Brazil [22].

A greater number of publications was observed beginning in 2017, with 2021 having the highest number of publications on compost barns with 26 publications in total. This large increase in publications indicates that the compost barn system has become a topic of global interest. The main topics discussed in that year were spatial variability in the thermal environment and bedding characteristics [23,24,25,26,27,28,29,30,31,32].

### 3.2. Relevant Publications and Characteristics of the Articles

The 20 most relevant articles were ranked by the number of citations, as shown in Table 1. The most cited study in the 15 years analyzed was written by Barberg et al. [5]. This result can be explained by the fact that these authors were pioneers in research on compost barns, and in the cited study, the authors aimed to describe the housing system, identify the management practices used for herds housed in compost barns, observe the welfare of cows, analyze herd performance and udder health before and after the change in the housing system and measure the producer’s satisfaction with the system; the authors collected data using direct observations of cows and their environments, examined DHIA (National Dairy Herd Information Association) records when available and evaluated historical information about milk tanks of milk processors when possible. In this study, the authors found an improvement in the comfort and longevity of the animals, as they observed a lower prevalence of hock injuries and lameness, lower rates of infection by mastitis, improvements in reproductive performance, and ease of completing daily tasks compared to other types of housing in addition to great satisfaction among the producers after changing to the compost barn housing system. In addition, they also observed that special attention to the procedures used to prepare cows at the time of milking is necessary to achieve satisfactory milk quality in this type of housing. The authors suggested that additional research is needed to address which alternative sources of litter can be used in this system.

In the second most cited study, Endres and Barberg [19] demonstrated that the compost barn system can be a suitable type of housing for dairy cows, since their observations of lying behavior, social interactions, and natural lying positions were not substantially different from those previously reported for other types of housing. Another important point addressed in the study was the importance of improving thermal comfort in dairy cattle facilities, which can optimize animal health and productivity, directing new research topics to improve this type of facility.

Klaas and Zadoks [33] discussed the fact that environmental mastitis is the most common and expensive form of mastitis in modern dairy herds. Thus, there is great pressure from producers and society to reduce the use of antibiotics as a tool for mastitis control. In addition, these authors provided an overview of the factors that influence the occurrence and control of environmental mastitis, proposing three priority areas for future research: (1) improved diagnostic tools for evidence-based guidance of antimicrobial treatment and transmission prevention measures; (2) tools to monitor and manage bacterial exposure in the dairy cow environment and host resistance to such exposure, for example, through the manipulation of cow microbiota; and (3) adequate communication strategies and socioeconomic incentives to increase acceptance by veterinarians and farmers and promote the adoption of existing and new mastitis control tools.

Lobeck et al. [34] investigated animal welfare by comparing conventional free-stall facilities with natural ventilation (NV) with free-stall facilities with cross ventilation (CV) and compost barn facilities (CB). The authors found that among the two free-stall housing options, CV improved the comfort indices of the cows compared to those in the NV facilities and that although the cows in CB facilities had better foot and leg health, as indicated by reductions in the prevalence of lameness and hock injuries, acquiring bedding and handling the bedding material may limit its use. However, the animals housed in CB facilities did not differ statistically in body condition, respiration rates, prevalence of mastitis, culling or mortality compared to animals housed in free-stall facilities with CV or NV.

Black et al. [4] characterized herd performance, the satisfaction and recommendations of the producers, and the management practices used by dairy farmers in the state of Michigan (USA) who adopted the compost barn system. The authors report that the performance of the compost barn system relies on meticulous management by producers, including adequate aeration, addition of litter, space per cow, and ventilation, ensuring the comfort and hygiene of the animals, which were the benefits most cited by the producers. Regarding herd performance, they reported increased milk production and reduced SCC, calving interval, and period of service after transitioning to compost barns. Conversely, the investment in the compost barn system was reduced compared to that in the free-stall system, although the variable cost associated with the litter material increased. However, the cost of litter can vary by region, making the best option dependent on the cost per cubic meter; after the litter is removed, it can be used as a hygienic and nutritious product in fields (i.e., as a fertilizer).

Until 2007, there were no publications on compost barn housing, and the current recommendations for design and management were based on the experiences of producers. Janni et al. [6] described recommendations for the layout and management of this system, being one of the first studies to do so.

Regarding the future of dairy housing, Bewley et al. [2] reviewed changes that were made over the last 100 years in relation to productivity, health, milk quality, reproduction, animal welfare, and farm profitability. The authors show that all housing systems are moving towards a system that allows greater comfort for cows and that the external pressure of dairy consumers and public perception may lead farmers to consider other alternatives to total confinement.

Leso et al. [35] reviewed the current scientific knowledge about compost barn housing to provide a comprehensive tool for producers and researchers using this housing system. In this review, the authors provide an overview of the reported benefits of the compost barn system relative to other types of housing for dairy cattle with regard to the well-being, hygiene, lameness, udder health, body condition, disposal rates, behavior, performance, and milk quality of the animals maintained in this system. In addition, they describe the system, including the design of the shed, alternative construction solutions, the management of and materials used for litter, and the quality and characteristics of the litter waste as an alternative for fertilizer and the costs involving the compost barn system.

To characterize the new sheds that were receiving much attention in prior years, Barberg et al. [18] conducted a descriptive study to describe the layout of the buildings, collect the dimensions of the buildings, characterize the bedding material used, and observe the management practices of the sheds that were used; as such, this is one of the pioneering studies in the research field.

Seeing the concern of producers regarding the availability of litter for compost barn sheds, especially sawdust, which is the most commonly used material, Shane et al. [20] conducted a descriptive study of the alternative litter materials used to partially or totally replace sawdust in composting barns used in dairy farms.

The main studies addressing the compost barn system were primarily focused on the welfare of dairy cows (Table 1), as evidenced by the descriptions of the layouts and management practices used in the compost barn system and the relationship between comfort-related bedding variables and better udder health and hygiene indices, lameness, and hock injuries.

### 3.3. Most Influential Journals

The main journals were classified by the number of citations (Table 2). Although there are variations in the specificities of these journals, we observed a predominance of journals focused on agricultural and biological sciences. The “Journal of Dairy Science” was highlighted, with more than three times the number of citations than the journal that ranks second, “Applied Engineering in Agriculture”. As this study is focused on the type of housing used for dairy cattle, this highlight can be explained by the extremely important role that the “Journal of Dairy Science” plays in the dissemination of scientific discoveries about the milk production chain worldwide and addressing existing and new technologies [46]. In addition, the “Journal of Dairy Science” is considered the main journal of general research on the milk production chain.

The second most relevant journal, “Applied Engineering in Agriculture”, may have been highlighted because the studies published in this journal are related to the layout and management of facilities. In addition, the journal published two of the first published studies on the compost barn system [6,18]. These articles are among the ten most relevant publications on this type of accommodation.

Table 2 shows a predominance of European journals; however, the two most relevant journals are from the United States, which is consistent with the origin of the compost barn system in this country, with the first compost barn dairy shed being built in Minnesota in 2001 [18]. Although “Transboundary and Emerging Diseases” and “Agronomy Research” have higher impact factors (JCR) than the “Journal of Dairy Science” and “Applied Engineering in Agriculture”, these journals do not have dairy cattle as their central theme, as is the case for the “Journal of Dairy Science” and “Livestock Science”, which may explain their placement in the ranking.

### 3.4. Author Publications

To identify the main authors of publications related to compost barns, the authors of studies with the highest numbers of citations were selected, as shown in Table 3. Subsequently, H index values were obtained from the Scopus and Web of Science databases to determine the impacts of the authors. The H index is defined as the number of articles with citations greater than or equal to a given number [47].

Of the 319 authors identified, the researcher Marcia I. Endres, from the Department of Animal Science of the University of Minnesota, was the author with the highest academic impact, with an H index of 22 (Scopus and Web of Science), 12 published documents, and 418 citations. Next, researcher Abby E. Barberg, also from the University of Minnesota, had an H index of 03 (Scopus and Web of Science), three published documents, and 215 citations (Table 3).

**Figure 3:**
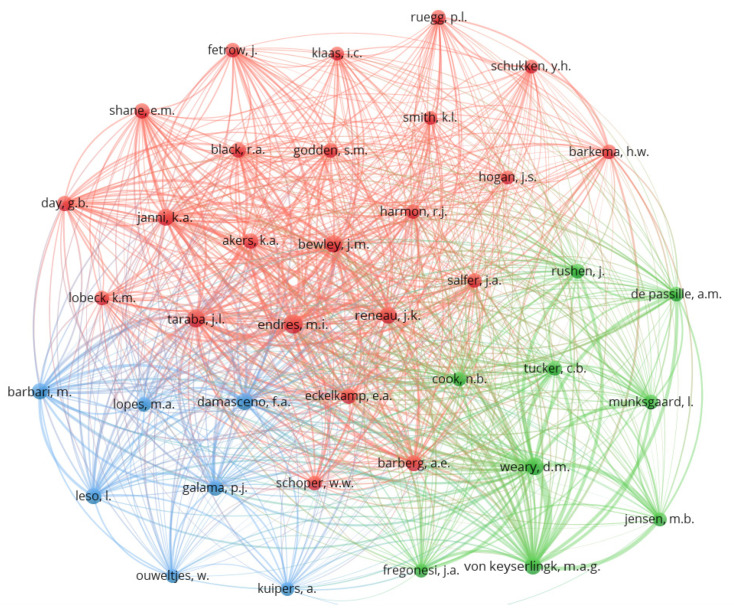
Scientific mapping of the co-citation of the most relevant authors in compost barn research.

To determine the relationships between the main authors with documents indexed in the Scopus and Web of Science databases, co-citations were mapped; authors with at least 30 citations were considered, which enabled the classification of the 40 authors shown in Figure 3.

The co-citation map illustrates the scientific network of a study based on the frequency with which two articles are cited together by a third document [48]. Each circle represents a reference (author); the size of the circle represents the influence of the author, and the color represents the area of knowledge (cluster) to which the study was grouped. Thus, it was possible to establish similarities, differences, relationships, and relevance among the authors who represent the intellectual basis of the compost barn system.

By analyzing the author co-citation network, three clusters were identified. The first cluster, in red, brings together eight of the top ten researchers, adding the greatest document value. The main focal areas are the study of animal zootechnical indices, animal welfare indicators, hygiene indices, lameness prevalence, and udder health after moving to the compost barn housing system. Comparisons between compost barns and open stall housing, a more conventional system, are often observed in this cluster, in addition to descriptive studies and characterization of the housing system, since these topics bring together the pioneering authors in research on compost barn housing.

Researchers in the green cluster have as their main focus the study of the relationship between environmental variables and stocking density of sheds and the effects on the laying behaviors and social interactions of cows housed in compost barns, aiming at greater animal welfare. Studies evaluating the prevalence of lameness and hock injuries and the effects of composting beds on these diseases are also included in this cluster.

The last cluster, in blue, is the smallest and includes studies focused mainly on the spatial distribution of environmental variables and the compost bed. In this cluster, studies involving costs, productivity, and animal health are also highlighted. Two of the prominent researchers of the compost barn system are found in this cluster. The use of geostatistics for the study of spatial variables is observed in this cluster.

### 3.5. Most Influential Countries

**Figure 4:**
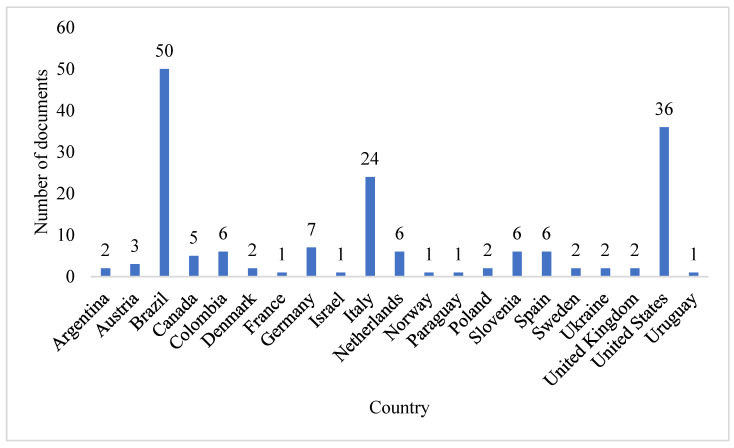
Number of publications by country.

Brazil and the United States are the largest producers of knowledge about the compost barn housing system. Brazil has the largest number of publications by country, as shown in Figure 4. However, the number of publications measures only the productivity of a country, institution or author and not the importance or impact of the studies, unlike the number of citations, which measures the total impact [49].

We can explain the large number of Brazilian studies by the implementation of compost barns in Brazil following American standards. However, due to the differences between regions, especially in the climate and management practices adopted by producers, it has been suggested that modifications in the initial recommendations may have been made by producers in Brazil and are continuously being implemented to adapt the system to different local conditions [50]. Thus, there is a great need for studies on implementing the compost barn system in Brazil to fully adapt the system to the climate in this country and maximize its potential.

**Figure 5:**
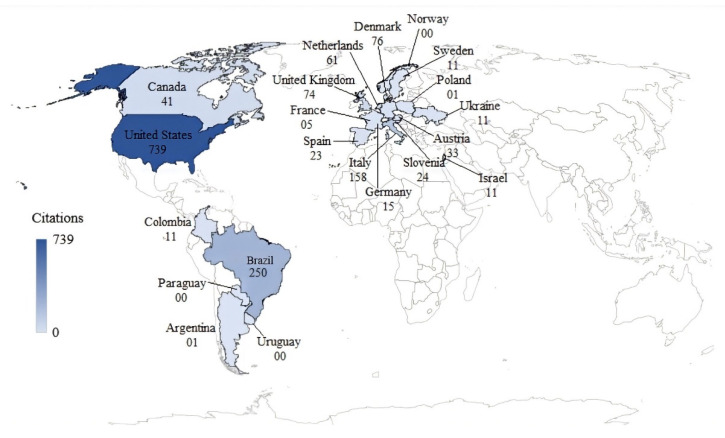
Number of citations by country.

Thus, the classification of countries according to the number of citations (Figure 5) allows us to classify the most relevant nations in this research field. Brazil and the United States are still the two most relevant countries. However, although Brazil has the largest number of publications, the United States is the country with the greatest impact on research on compost barns, with almost three times the number of citations as Brazil. After these two countries are Italy, Denmark, the United Kingdom, and The Netherlands.

The greater relevance of the United States can be explained by this country being the birthplace of the compost barn and publishing the first studies on this type of housing and most of the most relevant publications in addition to being the largest producer of milk globally. In Brazil, the compost barn system only began to be used in 2012 [51]. Today, there is widespread acceptance of this model in Brazil and worldwide due to the high degree of satisfaction of producers with its operation, as reported in several studies [4,5,44], which explains the growing number of publications from various countries.

### 3.6. Organizations Related to Research on the Compost Barn Housing System

Identifying the organizations responsible for the development of an area of knowledge is of fundamental importance in bibliometric analysis because it allows the determination of trends and relationships between these organizations [52].

**Figure 6:**
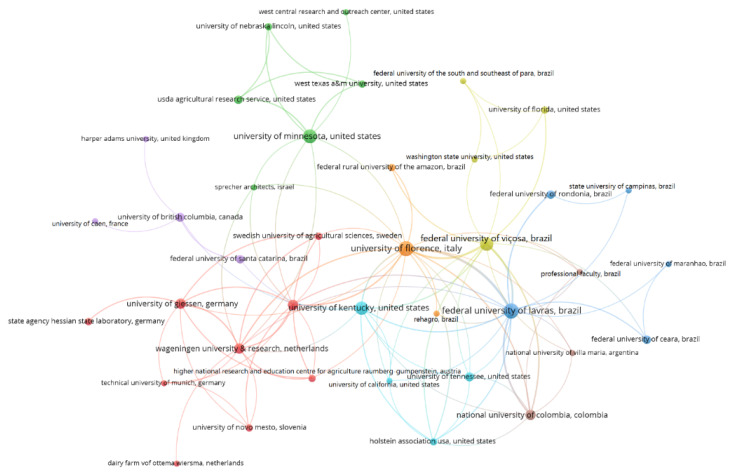
Scientific mapping network of educational and/or research organizations that produce knowledge about compost barns.

A total of 88 organizations were identified, 38 of which formed the largest network of interactions and the largest number of publications among the organizations identified and linked to the authors. The relationships between the scientific organizations that produced knowledge about compost barns are shown in Figure 6.

The main institutions involved in research on the compost barn system were divided into eight groups, and a large contribution of Brazilian universities to the development of research on the subject was observed: in the red group, the University of Giessen, the University of Ljubjana and Wageningen University & Research stood out; in the green group, the main institution was the University of Minnesota; in the dark blue group, the main institution was the Federal University of Lavras; in the yellow group, the main institution was the Federal University of Viçosa; in the purple group, the University of British Columbia and the Federal University of Santa Catarina were highlighted; in the light blue group, the main institution was the University of Michigan; in the orange group, the main institution was the University of Florence; and in the brown group, the National University of Colombia was highlighted.

The main institutions were the University of Firenze and the Federal University of Lavras, identified in the orange and dark blue regions of the map, respectively. On the map, these universities are linked directly or indirectly to virtually all the other institutions, showing strong international cooperation from these institutions. The emphasis on these two universities can be explained by both the large number of publications they have produced and the great cooperation in publishing between the two institutions (twelve co-authored publications). Studies from both institutions have focused on the spatial variability in thermal conditions and bedding variables related to animal thermal comfort.

Although these universities have great prominence in the network, the University of Minnesota (15 documents and 438 citations) and the University of Michigan (13 documents and 257 citations) are highly relevant to research on the compost barn system, since they have the highest and second highest numbers of citations, respectively. As the map was generated by evaluating the studies by using a measure of “weight” to relate the frequency and the relationship between the institutions, the Federal University of Lavras and the University of Florence had greater prominence due to their greater numbers of publications (21 documents and 129 citations and 20 documents and 133 citations, respectively). Five of the ten most highly cited authors (ENDRES, MI; BARBERG, AE; RENEAU, JK; JANNI, KA; and SHANE, EM) and pioneers in this research field are associated with the University of Minnesota, and publications from authors associated with this university are related to descriptions and management studies of compost barn housing. One of the ten most highly cited authors is associated with the University of Michigan (TARABA, JL), and the work associated with this institute is focused mainly on the variables that influence the composting bed.

Among the 38 organizations highlighted, eleven are Brazilian, and the most prominent are the Minas Gerais universities, the Federal University of Lavras and the Federal University of Viçosa, which can be explained by the role of Minas Gerais as the leader in national milk production, accounting for more than a quarter of national production [53].

The red group is formed exclusively by European institutions, and the fact that the University of Florence and the University of Caen, in the orange and purple groups, respectively, are the only European institutions included in other groups can be explained by these institutions following research guidelines. The research characterizing the red cluster mainly addresses the properties and cellular fractions of the bedding materials and their relationship with animal welfare, while that characterizing the purple cluster is more focused on factors associated with the prevalence of lameness and leg injuries in dairy cows.

### 3.7. Keywords Related to Compost Barn Research

Analysis of the co-occurrences between keywords seeks to determine when such terms occur together in a given sample, whether they appear in the title, in the abstract or in the list of keywords [54]. Thus, it is possible to evaluate the themes, trends, and research gaps in a given area. The larger the circle is, the higher the frequency of the term; the closer the circles are, the stronger the relationship between them.

In the map creation process, 306 keywords were identified. However, the software cannot differentiate words with different spellings or inflected words in the plural form from words in the singular form. To solve this issue, a thesaurus was created in the form of a *.txt* file and was used in VOSviewer so that these words were not read twice, leading to the generation of an erroneous map. When creating a map based on text data, a VOSviewer thesaurus file can be used to merge terms that are synonyms or present with different spellings and abbreviated terms with full terms or even to ignore terms [17]. The substitutions made using the thesaurus can be seen in Table 4.

**Figure 7:**
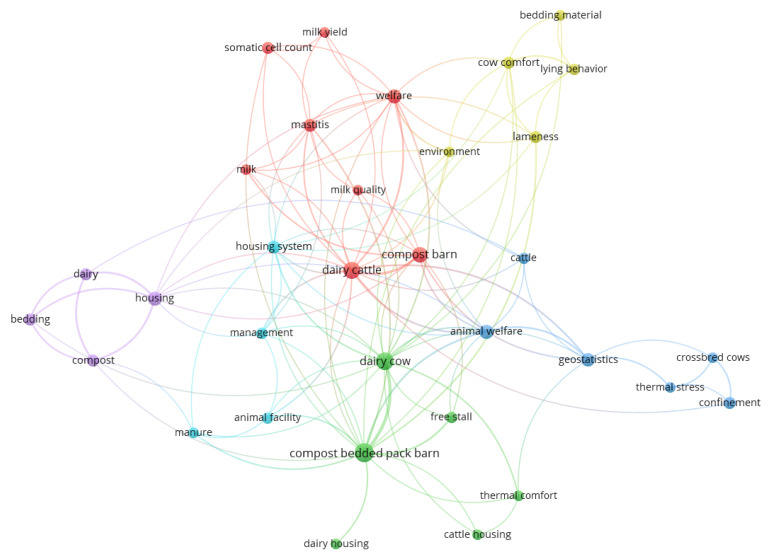
Network map between author keywords. Lines indicate co-occurrences between terms.

After eliminating this duplication of terms, 282 words were identified, and only the words with at least three occurrences were selected for the co-occurrence analysis of keywords used by the authors, the results of which are shown in Figure 7. As a result, the five keywords most frequent keywords were “compost bedded pack barn” (25 occurrences), “dairy cow” (19 occurrences), “dairy cattle” (16 occurrences), “compost barn” (13 occurrences), “animal welfare”, and “housing” (9 occurrences).

The figure shows six distinct groups: the red group predominantly represents studies on udder health and milk quality in compost barn housing; the green group includes a larger number of studies, focusing on comparative studies between compost barn systems and other types of housing; the dark blue group includes studies on the spatial variability in thermal conditions and bedding variables related to animal thermal comfort; the yellow group includes studies on litter properties and their relationship with productivity and animal welfare; the studies in the purple group are focused on the characterization and management of the compost barn system and the composting bed; and the studies in the light blue group focus on the potential for composting and litter management.

Among these groups, there is a strong focus on animal welfare, health, and productivity, which indicates a strong relationship between management focused on respect for the freedoms of animals and their high productivity and longevity. This map also contributes to the search for publications related to specific research areas related to compost barns and indicates how authors should organize their keywords to facilitate visualization.

### 3.8. Trends in Research on Compost Barns

**Figure 8:**
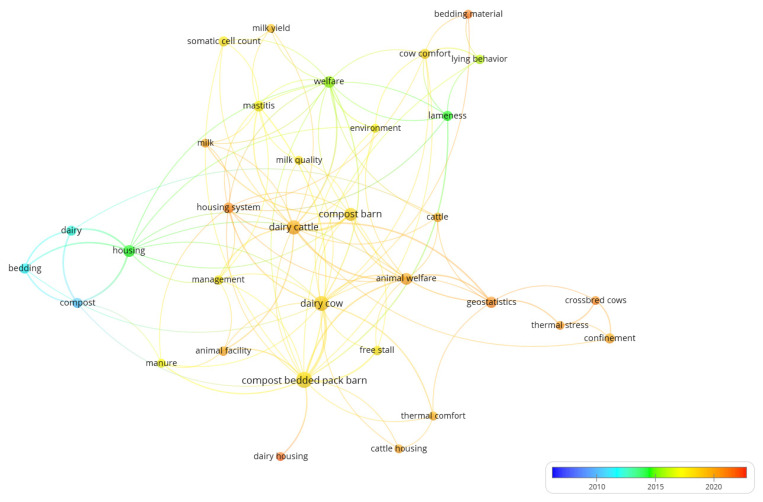
Map based on the co-occurrence of authors’ keywords and evolution from 2007 to 2022/April. The color scale represents the keyword’s year of dominance.

The publications included in this study followed trends according to the availability of knowledge about the system, use of technologies and increased requirements related to animal welfare and more efficient means of production. For better visualization, a map was created using a fractional counting method and overlap visualization, which gives scores to items and classifies them by color according to the score. The colors range from blue (lowest score) to orange and red (highest score). On the basis of bibliographic data on the co-occurrences of keywords associated with the authors, we determined the trends of use of these words over the last 15 years (Figure 8).

The information presented in Figure 7 allows us to characterize the three predominant groups by color. In the blue group, there was high proximity between the items; the green group focused on housing, while the yellow–orange group focused on compost bedded pack barns.

In the blue group, the use of keywords was closely linked to the theme of the first studies, which included descriptions of the types of accommodations used, since the research was still in the early stages. For this reason, we observed the prevalence of words such as “compost” and “bedding” linked to “dairy” between 2007 and mid-2012.

With some studies on this system already providing information on the layout and management of compost barns and the improvement in the indices of animal health associated with this housing type, we observed that in the green group, the central focus was on the relationship of the animals with the environment. This is demonstrated by the prevalence of terms such as “welfare”, “lying behaviour”, and “housing” between approximately 2014 and 2016.

More recently, in studies characterized by orange colors, there is a noticeable increase in the charge for animal welfare, since the “animal welfare” circle is more prominent than the “welfare” circle in the green group. The advancement of the use of technologies to study the environmental variables that influence the compost barn system is also clear, with the “geostatistics” circle being more prominent due to its warmer orange color.

Thus, this map confirms the trend of current research focusing on the spatial variables of sheds and their relationship with milk quality, heat stress, and animal welfare.

### 3.9. Pros and Cons of Compost Barns

According to the literature, the compost barn system presents some advantages compared to other traditional housing because it was thought to be healthier and provide more natural living conditions for the housed cows [19,35]. Some authors have found that the main benefits of this system include improved animal comfort, and natural behavior. Consequently, it could cause an improvement in milk production. Some studies have shown that high milk production levels comparable to free stalls are possible in compost barns [2,18,34,35].

Additionally, better reproductive rates, and reduction of hoof problems in dairy cows are reported in the literature [4,5,19,34]. Housed animals in compost barns have presented fewer hock lesions and lameness due to the lower exposure to concrete surfaces and injury-causing obstacles [2]. However, all of these advantages depend on correct system management, mainly when we think about the management of the bedded pack.

Producers must pay attention to maintaining adequate pack moisture, which is one of the essential factors in a compost barn [35], and especially difficult during the winter period in northern countries [1]. High pack moisture and inadequate composting of bedding materials are associated with the occurrence of dirty cows [55], cow mastitis risks [56], and the reduction of comfort and gaseous emission [35].

Lobeck et al. [34] point out that acquiring bedding and handling the bedding material may limit the use of compost-barn-type housing. The cost of the litter varies in different regions due to the low availability of materials [1,5] and the need for daily management of litter, which must be turned two to three times a day [6,9].

Wood shavings (sawdust) are the most used material in compost beds. However, due to low availability and increasing costs in some regions, it is a limiting factor. There are several studies involving the use of alternative materials as a substitute [20,21,30,57], which can be a determining factor in the expansion of the use of this system.

Regarding the economy, the investment in the compost barn system depends on the climate zone. It may be a lower construction cost system than other confinements due to the reduced area with concrete floor in (sub)tropical regions [6,7,35]. In humid continental climate and/or regions with less stable soil types, the needed larger concrete floor area and the larger roof construction require higher investments [1,35]. Moreover, the compost barn daily costs are usually higher, depending on the cost of obtaining the bedding material [1,58].

### 3.10. Limitations of a Bibliometric Analysis

The bibliometric analysis uses the application of quantitative techniques to bibliometric data, summarizing large amounts of data to present the state of intellectual structure and emerging trends in a topic or field of research [59]. Although this type of analysis efficiently summarizes and synthesizes data from the literature, it has some limitations.

The first limitation we can consider that this bibliometric analysis only considered manuscripts published in English languages. In this research, papers in different languages were considered, since they have an abstract or keywords in the English language.

The second limitation is that the obtained data from the databases are not exclusively produced for this type of analysis and may contain errors (such as duplicate data and erroneous entries) that will affect the analysis results. Thus, researchers should consider standardizing and structuring data to mitigate such errors [59]. This study observed a significant variation related to the authorship of the papers obtained from the two platforms (Scopus and Web of Science). The platform Scopus considers all authors of each manuscript, and Web of Science considers only one author. To evaluate the obtained information, the researcher needs to standardize the data obtained from both databases.

The third limitation is that the qualitative data can be quite subjective. The researcher needs to take care in making qualitative affirmations about the results and supplementing them with appropriate analyses [60].

Wallin [60] also points out that the bibliometric analysis can only offer a short-term forecast of the research field, and, therefore, the research should avoid making overly ambitious previews about the research field and its long-term impact.

Although there are these limitations, having a detailed knowledge of these limitations and performing rigorous data standardization, it is possible to efficiently interpret large amounts of data that can be very useful in the research field.

## 4. Conclusions

This bibliometric analysis allowed us to evaluate the scientific evolution and research, authorship, and citation patterns on the compost barn dairy farming system. From the results, it is possible to deduce current conditions and trends in the scientific research and publications in this field. The main countries, journals, institutions, researchers, and co-citation networks with the highest relevance to compost barn research were highlighted. Although there are some limitations to this type of analysis, it is possible to efficiently interpret large amounts of data, obtaining an overview to interpret a significant amount of data.

Based on the data, there was a significant increase in scientific publications on compost barn housing in the last 15 years (from 2007 to April 2022). The development of this system was driven mainly by research to improve animal welfare and productivity, reconciling a better animal comfort and lower incidence of injuries in animals with better hygiene, reproduction, and milk production levels, contributing to the growing demand and advances in food production and respect for animal freedoms. In addition, the development was driven by the need for more sustainable production; compost barns lead to improvements in the management of manure, which has potential agronomic value and may therefore return more profit to milk producers.

We can remark that the main benefits of the compost barn include improved animal comfort and natural behavior and its improvement in milk production. We can consider the better reproductive rates, and a reduction of hoof problems are found in this confinement system. However, to obtain good results with the compost barn system it is essential to maintain adequate pack moisture, otherwise dirty cows, mastitis, and problems with gaseous emission can occur.

It is important to mention that the financial investment in the compost barn depends on the climate zone. It may be a lower construction cost system than other confinements. Moreover, the compost barn daily costs are usually higher, depending on the cost of obtaining the bedding material that can vary in different regions due to the low availability of materials.

Among the most commonly used technologies in this type of housing, the use of geostatistics stands out. This tool has contributed to the development of analyses of the spatial variability in the thermal environment within compost barns, making it possible to make inferences and predictions from the data collected inside the barns.

The advancement of technologies applied in compost barn systems was demonstrated by mapping keywords used in studies published in the most important scientific journals. The main keywords used in the studies in recent years were “compost bedded pack barn”, “dairy cow”, “dairy cattle”, “compost barn”, “animal welfare”, and “housing”, which demonstrates strong trends in studies related to welfare and in the relationship between the animals and the environment or housing.

The development of this research field is mainly linked to milk-producing countries. The importance of the United States for the development of scientific knowledge about compost barns is evident, given that this country is the global leader in milk production. The Federal University of Lavras and the University of Florence have the highest numbers of publications, especially studies involving geostatistics and spatial variability in the thermal environment, coinciding with the large increase in the number of publications since 2018, which causes these institutions to have great prominence in research on compost barns.

Through the bibliometric analysis, it was possible to conclude that the compost barn system has become an increasingly popular and efficient system related to financial return and increasing the comfort, health, and quality of life of the animals housed in this system. However, since many countries have imported this technology from the United States without adapting it to their climatic conditions, studies regarding this type of production system must still be carried out to bring a better understanding of this system, in addition to providing results that are even more significant for these regions.

## Figures and Tables

**Table 1:** Top 20 scientific publications on compost barn from 2007 to 2022/April, sorted by citation number.

R	Title	Authors	PY	Journal	NC
1°	Performance and welfare of dairy cows in an alternative housing system in Minnesota	Barberg et al. [5]	2007b	Journal of Dairy Science	92
2°	Behavior of dairy cows in an alternative bedded-pack housing system	Endres and Barberg [19]	2007	Journal of Dairy Science	88
3°	An update on environmental mastitis: challenging perceptions	Klaas and Zadoks [33]	2018	Transboundary and Emerging Diseases	72
4°	Animal welfare in cross-ventilated, compost-bedded pack, and naturally ventilated dairy barns in the upper midwest	Lobeck et al. [34]	2011	Journal of Dairy Science	58
5°	Compost bedded pack dairy barn management, performance, and producer satisfaction	Black et al. [4]	2013	Journal of Dairy Science	58
6°	Compost dairy barn layout and management recommendations	Janni et al. [6]	2007	Applied Engineering in Agriculture	55
7°	A 100-year review: lactating dairy cattle housing management	Bewley et al. [2]	2017	Journal of Dairy Science	44
8°	Invited review: compost-bedded pack barns for dairy cows	Leso et al. [35]	2020	Journal of Dairy Science	38
9°	Compost dairy barns in Minnesota: a descriptive study	Barberg et al. [18]	2007a	Applied Engineering in Agriculture	35
10°	Alternative bedding materials for compost bedded pack barns in Minnesota: a descriptive study	Shane et al. [20]	2010a	Applied Engineering in Agriculture	33
11°	The relationship between compost bedded pack performance, management, and bacterial counts	Black et al. [36]	2014	Journal of Dairy Science	27
12°	Understanding compost bedded pack barns: interactions among environmental factors, bedding characteristics, and udder health	Eckelkamp et al. [37]	2016a	Livestock Science	27
13°	Fuzzy clustering and fuzzy validity measures for knowledge discovery and decision making in agricultural engineering	Mota et al. [38]	2018	Computers and Electronics in Agriculture	24
14°	Claw health and prevalence of lameness in cows from compost bedded and cubicle freestall dairy barns in Austria	Burgstaller et al. [39]	2016	Veterinary Journal	22
15°	Environmental characteristics and bacterial counts in bedding and milk bulk tank of low profile cross-ventilated, naturally ventilated, and compost bedded pack dairy barns	Lobeck et al. [40]	2012	Applied Engineering in Agriculture	21
16°	Sand bedded freestall and compost bedded pack effects on cow hygiene, locomotion, and mastitis indicators	Eckelkamp et al. [41]	2016b	Livestock Science	21
17°	Factors associated with mastitis epidemiologic indexes, animal hygiene, and bulk milk bacterial concentrations in dairy herds housed on compost bedding	Favero et al. [42]	2015	Livestock Science	19
18°	Prevalence of lameness and leg lesions of lactating dairy cows housed in southern Brazil: effects of housing systems	Costa et al. [43]	2018	Journal of Dairy Science	19
19°	A survey of Italian compost dairy barns	Leso et al. [44]	2013	Journal of Agricultural Engineering	18
20°	Effect of two housing systems on performance and longevity of dairy cows in northern Italy	Leso et al. [45]	2019	Agronomy Research	16

R: Ranking; PY: Publication Year and NC: Number of Citations.

**Table 2:** Top 6 sources of publications in the world on compost barn from 2007 to 2022/April.

R	Journal	SJR ^1^	CiteScore ^2^	JCR ^3^	H-i	ISSN	ND	NC
1°	Journal of Dairy Science	1.483	6.2	4.034	191	0022-0302	19	480
2°	Applied Engineering in Agriculture	0.276	1.9	0.985	54	0883-8542	6	158
3°	Livestock Science	0.622	2.9	1.943	111	1871-1413	5	85
4°	Transboundary And Emerging Diseases	1.392	7.6	5.005	63	1865-1674	1	72
5°	Agronomy Research	0.369	1.6	5.224	19	1406-894X	12	52
6°	Preventive Veterinary Medicine	0.816	4.1	2.67	95	0167-5877	3	26

^1^ Scopus Index; ^2^ Scopus Index; ^3^ Web of Science Index; H-i: H Index; ND: Number of documents and NC: Number of citations.

**Table 3:** Top ten relevant authors of publications on compost barn from 2007 to 2022/April.

R	Authors	Id.	H-i (Scopus)	H-i (WoS)	NC	ND
1°	Márcia I. Endres	Endres M.I.	22	22	418	12
2°	Abby E. Barberg	Barberg A.E.	3	3	215	3
3°	Jeffrey M. Bewley	Bewley J.M.	25	23	198	10
4°	Flávio Alves Damasceno	Damasceno F.A.	9	6	193	17
5°	Joseph L. Taraba	Taraba J.L.	15	10	179	9
6°	Jeffrey Kimball Reneau	Reneau J.K.	16	17	160	3
7°	Kevin A. Janni	Janni K.A.	22	18	144	4
8°	Matteo Barbari	Barbari M.	13	2	121	17
9°	Elizabeth A. Eckelkamp	Eckelkamp E.A.	6	4	108	5
10°		Shane E.M.	3	3	105	4

R: Ranking, H-i: H index, NC: Number of citations, ND: Number of documents.

**Table 4:** Dictionary of synonyms based on keywords found in publications.

Found Synonyms	Merge of Terms
Bedding material; Bedding materials	Bedding material
Compost barn; compost barns; system compost barn	Compost barn
Compost bedded pack; compost bedded pack barn; compost bedded pack barns; compost bedded-pack barns; compost-bedded pack; compost-bedded pack barn; compost-bedded pack barns	Compost bedded pack barn
Compost dairy barn; compost dairy barns	Compost dairy barn
Confinement; confinement housing	Confinement
Cow; cows	Cow
Dairy cow; dairy cows	Dairy cow
Free stall barn; freestall system; freestall; freestall barn	Free stall
Housing system; housing systems	Housing system
Hygiene and lameness scores; lameness and hygiene scores	Hygiene and lameness scores
Loose housing; loose housing system	Loose housing
Moisture; moisture content	Moisture
Nitrogen loss; nitrogen losses	Nitrogen loss
Production cost; production costs	Production cost
Somatic cell count; SCC	Somatic cell count
Spatial variability; spatial variability	Spatial variability

## Data Availability

Data sharing not applicable.
